# Dengue, chikungunya and Zika virus surveillance in blood donors in Brazil, 2019–2021

**DOI:** 10.1111/vox.70150

**Published:** 2025-12-17

**Authors:** Eduard Grebe, Renata Buccheri, Leilani Montalvo, Simone Kashima, Carolina Miranda, Pamela Milani, Mars Stone, Kristin Livezey, Ligia Capuani, Cecilia S. Alencar, Luiz Amorim, Paula Loureiro, Maisa Ribeiro, Allyson Guimarães da Costa, Alfredo Mendrone, Michael P. Busch, Ester C. Sabino, Brian Custer, Recipient Epidemiology and Donor Evaluation Study‐IV‐Pediatric (REDS‐IV‐P) Brazil

**Affiliations:** ^1^ Vitalant Research Institute San Francisco California USA; ^2^ SACEMA, Centre for Epidemic Response and Innovation (CERI) Stellenbosch University Stellenbosch South Africa; ^3^ Department of Epidemiology and Biostatistics University of California, San Francisco San Francisco California USA; ^4^ Fundação Hemocentro de Ribeirão Preto São Paulo São Paulo Brazil; ^5^ Programa de Pós‐Graduação em Ciências da Saúde: Infectologia e Medicina Tropical, Faculdade de Medicina Universidade Federal de Minas Gerais Belo Horizonte Minas Gerais Brazil; ^6^ Department of Laboratory Medicine University of California San Francisco San Francisco California USA; ^7^ Grifols Diagnostics San Diego California USA; ^8^ Instituto de Medicina Tropical, Faculdade de Medicina Universidade de São Paulo São Paulo São Paulo Brazil; ^9^ LIM‐03 Laboratório de Medicina Laboratorial, Hospital das Clínicas, Faculdade de Medicina Universidade de São Paulo São Paulo São Paulo Brazil; ^10^ Fundação Hemominas Belo Horizonte Minas Gerais Brazil; ^11^ Fundação Hemorio Rio de Janeiro Rio de Janeiro Brazil; ^12^ Fundação Hemope Recife Pernambuco Brazil; ^13^ Fundação Hospitalar de Hematologia e Hemoterapia do Amazonas Manaus Amazonas Brazil; ^14^ Universidade Federal do Amazonas Manaus Amazons Brazil; ^15^ Fundação Pro‐Sangue Homcentro de São Paulo São Paulo Brazil; ^16^ Departamento de Patologia, Faculdade de Medicina Instituto de Medicina Tropical, Faculdade de Medicina Universidade de São Paulo São Paulo São Paulo Brazil

**Keywords:** arbovirus, blood donors, chikungunya virus, dengue virus, surveillance, Zika virus

## Abstract

**Background and Objectives:**

Arbovirus infections are a major public health concern in Brazil and an ongoing blood safety concern. Periodic outbreaks of such infections are common in the general population. This study aimed to establish the rates of dengue, chikungunya and Zika virus (DENV, CHIKV, ZIKV) RNAemia among blood donors at six public blood centres during the 2019–2020 and 2020–2021 outbreak seasons.

**Materials and Methods:**

Residual minipool samples from nucleic acid testing (NAT) screening for human immunodeficiency virus, hepatitis B virus and hepatitis C virus were further pooled to form pools of 18 donation samples and tested using a Grifols research‐use‐only triplex transcription‐mediated amplification assay for DENV, CHIKV and ZIKV RNA to establish the rates of RNAemia and infection incidence. We used these rates and estimated the durations of RNAemia, juxtaposed with public health reporting of cases.

**Results:**

A total of 5,616 minipools representing 101,088 donations were tested. During both outbreak seasons, the highest rates of DENV RNAemia were observed in Ribeirão Preto, at 122/100,000 donations (95% confidence interval [CI]: 66–224) and 100/100,000 (95% CI: 52–189), respectively, with DENV RNAemia also detected in São Paulo, Recife and Manaus in the 2019/2020 season and in the latter two during the 2020/2021 season. CHIKV RNAemia was detected in Recife and ZIKV RNAemia in Manaus during the 2020/2021 season. The estimated numbers of DENV, CHIKV and ZIKV RNAemic components released for transfusion over the study period were 338, 22 and 6, respectively.

**Conclusion:**

Surveillance for arbovirus RNAemia in blood donors is a useful adjunct to public health surveillance, particularly when surveillance systems are under strain, and has implications for transfusion safety.

**Highlights**

- Arbovirus infections are a public health and ongoing blood safety concern in Brazil, including during major public health crises such as the COVID‐19 pandemic.
- Residual blood donation screening samples were tested using a multiplexed NAT assay for dengue, chikungunya and Zika virus (DENV, CHIKV and ZIKV) RNA, and the rates of RNAemia in asymptomatic Brazilian blood donors were estimated during two arbovirus outbreak seasons.
- Surveillance for arbovirus infections in blood donors is a useful adjunct to public health surveillance, with this study documenting substantial rates of DENV infection and low rates of CHIKV infection in blood donors, as well as release of RNAemic blood components, representing potential risks to transfusion recipients.

## INTRODUCTION

Arbovirus infections are a major public health concern in Brazil. Disease incidence typically varies seasonally, with substantial year‐to‐year differences. Transmission is most common during November to March, when higher temperatures and precipitation favour vector proliferation throughout much of the country. Arboviruses that cause asymptomatic short‐duration viraemia and periodic outbreaks with high incidence in the general population, such as the dengue, chikungunya and Zika viruses (DENV, CHIKV, ZIKV), represent an ongoing concern for blood safety worldwide. DENV and other arboviruses have been demonstrated to be transfusion‐transmissible [1]. These concerns are particularly acute in Latin America and other regions where autochthonous spread occurs [2]. Further, sporadic and seasonal outbreaks of arbovirus infections make policies for donor screening challenging and variable from country to country. Multicentre studies of the risks associated with asymptomatic arbovirus infections in blood donors in Brazil are uncommon.

In this study, we utilized nucleic acid testing (NAT) for DENV, CHIKV and ZIKV RNA in residual samples from blood donors to establish the rates of RNAemia and estimate the incidence of infection in blood donors at six public blood centres participating in Phase 1 of the Recipient Epidemiology and Donor Evaluation Study‐IV‐Pediatric (REDS‐IV‐P) Brazil before the start and in the first year of the COVID‐19 pandemic.

## MATERIALS AND METHODS

The study focused on two 5‐month periods (November–March), coinciding with the typical arbovirus outbreak season in the years 2019–2021. Blood donor samples were collected at six blood centres: Fundação Pro‐Sangue (FPS) in São Paulo, Fundação Hemocentro de Ribeirão Preto (Hemoribeirão), Hemominas in Belo Horizonte, Hemorio in Rio de Janeiro, Hemope in Recife and Fundação Hemoam in Manaus, and tested for DENV, CHIKV and ZIKV RNA.

In public blood centres in Brazil, donations are screened for human immunodeficiency virus, hepatitis B virus and hepatitis C virus  using NAT on pooled plasma samples consisting of minipools of six donations (MP6). During the study period, residual MP6 samples were recovered after NAT screening and further pooled to create MP18 pools on a weekly basis. Occasionally, MP12 pools were created when insufficient samples were available. Samples were not available for November and December 2019 at Hemoam. Samples were shipped frozen from Brazil and tested at the Vitalant Research Institute (San Francisco, CA) using a research‐use‐only Grifols triplex DENV, CHIKV and ZIKV real‐time prototype transcription mediated amplification assay (Grifols Diagnostic Solutions, San Diego, CA) on the Panther platform. Its performance is comparable to that of the multiplexed, CE‐marked Grifols Procleix ArboPlex assay, for which analytical sensitivities of <10 IU/mL for ZIKV and CHIKV and <20 RNA copies/mL for DENV serotypes I–IV have been reported [3], and for DENV detection comparable to that of the CE‐marked Grifols Procleix Dengue Virus assay (analytical sensitivities of <30 copies/mL for the four serotypes) [4].

Virus‐specific prevalence of RNAemia, that is, the proportion of donations reactive for viral RNA, was estimated (per 100,000 donations) by month and overall, for each arbovirus outbreak season, using the method of Hepworth and Biggerstaff for pooled sample testing with variable pool sizes [5], with 95% skewness‐corrected score confidence intervals (CIs). The incidence of infection in blood donors was estimated by month and outbreak season using a previously described method [4], based on published estimates of the duration of RNAemia for each virus [6, 7, 8]. To juxtapose the prevalence of RNAemia and infection incidence in donors with public health surveillance, the numbers of dengue, chikungunya and Zika cases reported to public health authorities were obtained from the Brazilian national disease notification system (DATASUS TABNET, available at http://tabnet.datasus.gov.br). Rates per 100,000 population were calculated using annual population estimates for the regions serviced by each blood centre (Brazilian Institute of Geography and Statistics [IBGE], available at https://www.ibge.gov.br/).

The number of RNAemic components released for transfusion was estimated by multiplying the prevalence of RNAemia with the number of donations collected by each blood centre during a given time period, and by the average number of components produced from each whole blood donation in Brazil (2.3) [9].

This study was approved by the Brazilian National Ethical Committee (protocol number 53543316.3.1001.0065), the local ethics committees of each participating centre and the Institutional Review Boards at the University of California, San Francisco, and Westat.

## RESULTS

A total of 5,616 minipools, comprising 101,088 donation samples, were tested. During the 2019/2020 outbreak season, the highest rate of DENV RNAemia was in Hemoribeirão, at 122.2/100,000 donations (95% CI: 66.4–224.4), followed by Hemoam at 56.9/100,000 (95% CI: 19.3–166.6). DENV RNAemia was also detected at FPS and Hemope (Table 1). No CHIKV or ZIKV RNAemia was observed during the first outbreak season at any blood centre. During the 2020/2021 outbreak season, the highest rate of DENV RNAemia was also at Hemoribeirão, at 99.6/100,000 (95% CI: 52.4–188.9), followed by Hemoam at 44.6/100,000 (95% CI: 17.3–114.4) and Hemope at 34/100,000 (95% CI: 11.6–99.8). CHIKV RNAemia was detected at Hemope, at a rate of 22.7/100,000 (95% CI: 6.2–82.4), and ZIKV RNAemia at Hemoam, at a rate of 11.1/100,000 (95% CI: 2.0–62.8). No arbovirus RNAemia was detected at Hemorio and Hemominas in either season. The number of samples and donations tested by month and rates of RNAemia by month are shown in Table S1.

**TABLE 1:** Prevalence of RNAemic donations, by arbovirus outbreak season.

Blood centre	Nov 2019–Mar 2020 RNAemic donations per 100,000 (95% CI)			Nov 2020–Mar 2021 RNAemic donations per 100,000 (95% CI)		
	DENV	CHIKV	ZIKV	DENV	CHIKV	ZIKV
Fundação Pro‐Sangue, São Paulo	22.4 (6.2–81.6)	0 (0–42.9)	0 (0–42.9)	0 (0–42)	0 (0–42)	0 (0–42)
Hemoribeirão, Ribeirão Preto	122.2 (66.4–224.4)	0 (0–46.3)	0 (0–46.3)	99.6 (52.4–188.9)	0 (0–42)	0 (0–42)
Hemominas, Belo Horizonte	0 (0–45.4)	0 (0–45.4)	0 (0–45.4)	0 (0–43.6)	0 (0–43.6)	0 (0–43.6)
Hemorio, Rio de Janeiro	0 (0–46)	0 (0–46)	0 (0–46)	0 (0–43.9)	0 (0–43.9)	0 (0–43.9)
Hemope, Recife	12 (2.1–67.9)	0 (0–46)	0 (0–46)	34 (11.6–99.8)	22.7 (6.2–82.4)	0 (0–43.3)
Hemoam, Manaus^a^	56.9 (19.3–166.6)	0 (0–72.1)	0 (0–72.1)	44.6 (17.3–114.4)	0 (0–42.5)	11.1 (2–62.8)

Abbreviations: CHIKV, chikungunya virus; CI, confidence interval; DENV, dengue virus; ZIKV, Zika virus.

^a^ Data for November and December 2019 were unavailable.

**FIGURE 1:**
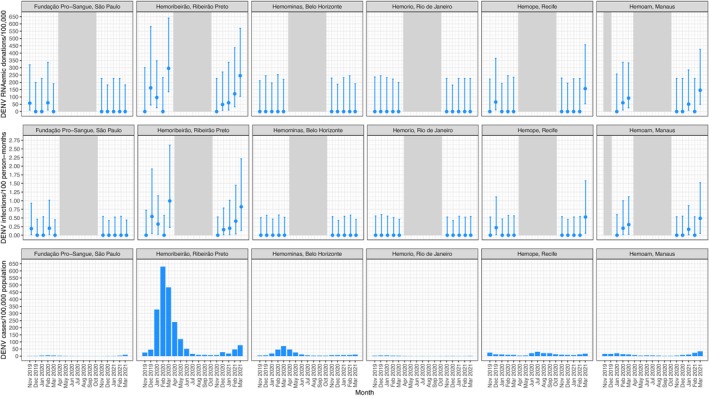
Prevalence of dengue virus (DENV) RNAemic donations, incidence of infections in blood donors and reported case rates at six Brazilian blood centres.

Incidence in donors paralleled RNAemia rates, with the highest estimated DENV incidence during both outbreak seasons at Hemoribeirão. Monthly incidence reached a peak of 0.99 infections per 100 person‐months (PM) (95% CI: 0.22–2.61) in March 2020 during the first period, and of 0.82 infections per 100 PM (95% CI: 0.14–2.22) in March 2021 during the second period. The prevalence of DENV RNAemia, infection incidence in donors and reported cases per 100,000 population are shown for each blood centre by month in Figure 1. The prevalence of CHIKV RNAemia, donor incidence and reported case rates are shown by blood centre and month in Figure S1 and those of ZIKV in Figure S2. Monthly incidence in blood donors and reported clinical case rates are provided in Table S2.

The estimated number of DENV RNAemic components released for transfusion was 184 during the 2019/2020 outbreak season and 154 during the 2020/2021 outbreak season. An estimated 22 CHIKV RNAemic components and 6 ZIKV RNAemic components were released, all during 2020/2021. None of the participating blood centres used pathogen reduction during the study period (Table 2).

**TABLE 2:** RNAemic donations and blood components, by arbovirus outbreak season.

Blood centre	Nov 2019–Mar 2020 donations (components)			Nov 2020–Mar 2021 donations (components)		
	DENV	CHIKV	ZIKV	DENV	CHIKV	ZIKV
Fundação Pro‐Sangue, São Paulo	11 (24)	0 (0)	0 (0)	0 (0)	0 (0)	0 (0)
Hemoribeirão, Ribeirão Preto	60 (133)	0 (0)	0 (0)	47 (105)	0 (0)	0 (0)
Hemominas, Belo Horizonte	0 (0)	0 (0)	0 (0)	0 (0)	0 (0)	0 (0)
Hemorio, Rio de Janeiro	0 (0)	0 (0)	0 (0)	0 (0)	0 (0)	0 (0)
Hemope, Recife	5 (12)	0 (0)	0 (0)	12 (27)	10 (22)	0 (0)
Hemoam, Manaus	7 (15)	0 (0)	0 (0)	10 (22)	0 (0)	3 (6)
Total	83 (184)	0 (0)	0 (0)	69 (154)	10 (22)	3 (6)

Abbreviations: CHIKV, chikungunya virus; DENV, dengue virus; ZIKV, Zika virus.

## DISCUSSION

This study provides evidence of pre‐symptomatic or asymptomatic dengue infection among blood donors, who were RNAemic at the time of donation, in São Paulo, Ribeirão Preto, Recife and Manaus during the 2019/2020 arbovirus outbreak season, and in the latter three cities also during the 2020/2021 season. The prevalence of DENV RNAemia in Ribeirão Preto was high in both periods (≥1 per 1000 donations), exceeding the magnitude observed in our previous study at four blood centres during 2016–2019, with the exception of the November 2018–June 2019 period in Belo Horizonte when a prevalence of ~3.6 per 1000 donations was observed [4]. Public health reporting of dengue cases in Ribeirão Preto during the first period suggests a large epidemic, with over 300 cases per 100,000 population reported during January, February and March 2020, with a peak of over 600 cases per 100,000 in February. The highest estimated monthly incidence, in March 2020, was about 1%. The prevalence of RNAemia and incidence in our study during the second period were not accompanied by similarly high case rates (Figure 1). In the other cities during this study period, public health reporting of dengue cases never exceeded 100 cases per 100,000 population per month.

Almost no evidence of ZIKV infection in donors was found during this study period. ZIKV RNA was detected only in March 2021 at Hemoam, at <50/100,000 donations (Figure S2). Although low rates of Zika cases were reported throughout the study period in all regions, it is possible these cases were not laboratory‐confirmed and could have been infections with other pathogens, including DENV. Our findings are consistent with other epidemiological data, which documented a steep decline in Zika incidence in Brazil after the 2015–2016 epidemic [10].

Transfusion‐transmitted arbovirus infection has been a blood safety concern, particularly after documentation of transfusion‐transmitted West Nile virus. Transfusion‐transmitted chikungunya remains a theoretical concern [2], but our study demonstrated that CHIKV RNAemic components were released for transfusion, representing a potential risk to the recipients. A study of transfusion‐transmitted dengue at Hemope and Hemorio showed more than one‐third risk of transmission after transfusing components derived from viraemic donors but did not show additional clinical symptoms of dengue in the recipients [11]. During our previous study, it was estimated that >300 DENV RNAemic components were released, but it is not known whether the RNAemia represents viraemia capable of causing infection in recipients. This is also a limitation of our study. Additional studies are needed to better understand the risk that RNAemic blood components pose to transfusion recipients, including chronically transfused individuals. A further limitation is that our estimates of the number of RNAemic components released do not account for factors such as infectious markers and discarded outdated components.

The two arbovirus outbreak seasons assessed in this study coincided with the onset of the COVID‐19 pandemic in early 2020. Widespread public health interventions, such as mobility restrictions and social distancing, were in place during the second outbreak season [12]. These measures likely contributed to a decline in arbovirus transmission by limiting human movement and reducing the geographic spread of outbreaks. At the same time, constrained healthcare access, reduced diagnostic capacity and the redirection of public health resources to the COVID‐19 response likely led to underreporting of arbovirus cases. Despite these disruptions, our findings show continued detection of dengue RNAemia in blood donors during the second outbreak season, particularly in Hemoribeirão. Notably, this ongoing arbovirus circulation was not reflected in public health clinical case reports. These findings demonstrate the value of blood donor surveillance for arbovirus RNAemia as an adjunct to public health case surveillance when routine surveillance systems are under strain or facing resource constraints.

## CONFLICT OF INTEREST STATEMENT

K.L. is an employee of Grifols Diagnostic Solutions. Vitalant Research Institute receives research support from Grifols Diagnostic Solutions. The authors declare no other conflicts of interest.

## Supporting information


**Table S1.** Minipool samples and donations tested, and prevalence of RNAemic donations by month.
**Table S2.** Incidence of infection in blood donors and reported case rates by month.
**Figure S1.** Prevalence of chikungunya virus RNAemic donations, incidence of infections in blood donors and reported case rates at six Brazilian blood centres.
**Figure S2.** Prevalence of Zika virus RNAemic donations, incidence of infections in blood donors and reported case rates at six Brazilian blood centres.

## Data Availability

The data that support the findings of this study are available from the corresponding author upon reasonable request.
