# Supplementary material for: Dengue, chikungunya and Zika virus surveillance in blood donors in Brazil, 2019–2021
Source: Vox Sang. 2025 Dec 17;121(2):196–201. doi: 10.1111/vox.70150 (PMC12890435; doi:10.1111/vox.70150)

# Supplementary Materials

# Grebe, E. *et al.* Dengue, Chikungunya, and Zika Virus Surveillance in Blood Donors in Brazil, 2019-2021.

Supplementary Table 1. Minipool samples and donations tested, and prevalence of RNAemic donations by month

| **Hemocenter** | **Month** | **MPs tested** | **Donations tested** | **RNAemic donations / 100,000 (95% CI)** | | | |
| --- | --- | --- | --- | --- | --- | --- | --- |
|  |  |  |  | **DENV** | **CHIKV** | **ZIKV** |  |
| Fundação Pro-Sangue, São Paulo | November 2019 | 97 | 1746 | 57 (10-320) | 0 (0-216) | 0 (0-216) |  |
| Fundação Pro-Sangue, São Paulo | December 2019 | 105 | 1890 | 0 (0-199) | 0 (0-199) | 0 (0-199) |  |
| Fundação Pro-Sangue, São Paulo | January 2020 | 92 | 1656 | 0 (0-227) | 0 (0-227) | 0 (0-227) |  |
| Fundação Pro-Sangue, São Paulo | February 2020 | 92 | 1656 | 60 (11-337) | 0 (0-227) | 0 (0-227) |  |
| Fundação Pro-Sangue, São Paulo | March 2020 | 110 | 1980 | 0 (0-191) | 0 (0-191) | 0 (0-191) |  |
| Fundação Pro-Sangue, São Paulo | November 2020 | 92 | 1656 | 0 (0-227) | 0 (0-227) | 0 (0-227) |  |
| Fundação Pro-Sangue, São Paulo | December 2020 | 115 | 2070 | 0 (0-182) | 0 (0-182) | 0 (0-182) |  |
| Fundação Pro-Sangue, São Paulo | January 2021 | 92 | 1656 | 0 (0-227) | 0 (0-227) | 0 (0-227) |  |
| Fundação Pro-Sangue, São Paulo | February 2021 | 92 | 1656 | 0 (0-227) | 0 (0-227) | 0 (0-227) |  |
| Fundação Pro-Sangue, São Paulo | March 2021 | 115 | 2070 | 0 (0-182) | 0 (0-182) | 0 (0-182) |  |
| Hemoribeirão, Ribeirão Preto | November 2019 | 69 | 1242 | 0 (0-301) | 0 (0-301) | 0 (0-301) |  |
| Hemoribeirão, Ribeirão Preto | December 2019 | 69 | 1242 | 162 (45-582) | 0 (0-301) | 0 (0-301) |  |
| Hemoribeirão, Ribeirão Preto | January 2020 | 116 | 2088 | 96 (26-347) | 0 (0-181) | 0 (0-181) |  |
| Hemoribeirão, Ribeirão Preto | February 2020 | 90 | 1620 | 0 (0-232) | 0 (0-232) | 0 (0-232) |  |
| Hemoribeirão, Ribeirão Preto | March 2020 | 115 | 2070 | 296 (136-640) | 0 (0-182) | 0 (0-182) |  |
| Hemoribeirão, Ribeirão Preto | November 2020 | 92 | 1656 | 0 (0-227) | 0 (0-227) | 0 (0-227) |  |
| Hemoribeirão, Ribeirão Preto | December 2020 | 115 | 2070 | 48 (9-271) | 0 (0-182) | 0 (0-182) |  |
| Hemoribeirão, Ribeirão Preto | January 2021 | 92 | 1656 | 60 (11-337) | 0 (0-227) | 0 (0-227) |  |
| Hemoribeirão, Ribeirão Preto | February 2021 | 92 | 1656 | 121 (33-437) | 0 (0-227) | 0 (0-227) |  |
| Hemoribeirão, Ribeirão Preto | March 2021 | 115 | 2070 | 246 (105-570) | 0 (0-182) | 0 (0-182) |  |
| **Hemocenter** | **Month** | **MPs tested** | **Donations tested** | **RNAemic donations / 100,000 (95% CI)** | | | |
|  |  |  |  | **DENV** | **CHIKV** | **ZIKV** |  |
| Hemominas, Belo Horizonte | November 2019 | 99 | 1782 | 0 (0-211) | 0 (0-211) | 0 (0-211) |  |
| Hemominas, Belo Horizonte | December 2019 | 85 | 1530 | 0 (0-245) | 0 (0-245) | 0 (0-245) |  |
| Hemominas, Belo Horizonte | January 2020 | 107 | 1926 | 0 (0-196) | 0 (0-196) | 0 (0-196) |  |
| Hemominas, Belo Horizonte | February 2020 | 82 | 1476 | 0 (0-254) | 0 (0-254) | 0 (0-254) |  |
| Hemominas, Belo Horizonte | March 2020 | 95 | 1710 | 0 (0-220) | 0 (0-220) | 0 (0-220) |  |
| Hemominas, Belo Horizonte | November 2020 | 91 | 1638 | 0 (0-229) | 0 (0-229) | 0 (0-229) |  |
| Hemominas, Belo Horizonte | December 2020 | 112 | 2016 | 0 (0-187) | 0 (0-187) | 0 (0-187) |  |
| Hemominas, Belo Horizonte | January 2021 | 90 | 1620 | 0 (0-232) | 0 (0-232) | 0 (0-232) |  |
| Hemominas, Belo Horizonte | February 2021 | 85 | 1530 | 0 (0-245) | 0 (0-245) | 0 (0-245) |  |
| Hemominas, Belo Horizonte | March 2021 | 110 | 1980 | 0 (0-191) | 0 (0-191) | 0 (0-191) |  |
| Hemorio, Rio de Janeiro | November 2019 | 88 | 1584 | 0 (0-237) | 0 (0-237) | 0 (0-237) |  |
| Hemorio, Rio de Janeiro | December 2019 | 85 | 1530 | 0 (0-245) | 0 (0-245) | 0 (0-245) |  |
| Hemorio, Rio de Janeiro | January 2020 | 90 | 1620 | 0 (0-232) | 0 (0-232) | 0 (0-232) |  |
| Hemorio, Rio de Janeiro | February 2020 | 94 | 1692 | 0 (0-222) | 0 (0-222) | 0 (0-222) |  |
| Hemorio, Rio de Janeiro | March 2020 | 105 | 1890 | 0 (0-199) | 0 (0-199) | 0 (0-199) |  |
| Hemorio, Rio de Janeiro | November 2020 | 92 | 1656 | 0 (0-227) | 0 (0-227) | 0 (0-227) |  |
| Hemorio, Rio de Janeiro | December 2020 | 116 | 2088 | 0 (0-181) | 0 (0-181) | 0 (0-181) |  |
| Hemorio, Rio de Janeiro | January 2021 | 92 | 1656 | 0 (0-227) | 0 (0-227) | 0 (0-227) |  |
| Hemorio, Rio de Janeiro | February 2021 | 92 | 1656 | 0 (0-227) | 0 (0-227) | 0 (0-227) |  |
| Hemorio, Rio de Janeiro | March 2021 | 92 | 1656 | 0 (0-227) | 0 (0-227) | 0 (0-227) |  |
| **Hemocenter** | **Month** | **MPs tested** | **Donations tested** | **RNAemic donations / 100,000 (95% CI)** | | | |
|  |  |  |  | **DENV** | **CHIKV** | **ZIKV** |  |
| Hemope, Recife | November 2019 | 94 | 1692 | 0 (0-222) | 0 (0-222) | 0 (0-222) |  |
| Hemope, Recife | December 2019 | 85 | 1530 | 65 (12-365) | 0 (0-245) | 0 (0-245) |  |
| Hemope, Recife | January 2020 | 109 | 1962 | 0 (0-192) | 0 (0-192) | 0 (0-192) |  |
| Hemope, Recife | February 2020 | 85 | 1530 | 0 (0-245) | 0 (0-245) | 0 (0-245) |  |
| Hemope, Recife | March 2020 | 89 | 1602 | 0 (0-235) | 0 (0-235) | 0 (0-235) |  |
| Hemope, Recife | November 2020 | 91 | 1638 | 0 (0-229) | 0 (0-229) | 0 (0-229) |  |
| Hemope, Recife | December 2020 | 108 | 1944 | 0 (0-194) | 0 (0-194) | 0 (0-194) |  |
| Hemope, Recife | January 2021 | 93 | 1674 | 0 (0-225) | 60 (11-334) | 0 (0-225) |  |
| Hemope, Recife | February 2021 | 92 | 1656 | 0 (0-227) | 60 (11-337) | 0 (0-227) |  |
| Hemope, Recife | March 2021 | 107 | 1926 | 157 (54-457) | 0 (0-196) | 0 (0-196) |  |
| Hemoam, Manaus | November 2019 | 81 | 1458 | 0 (0-257) | 0 (0-257) | 0 (0-257) |  |
| Hemoam, Manaus | December 2019 | 92 | 1656 | 61 (11-337) | 0 (0-227) | 0 (0-227) |  |
| Hemoam, Manaus | January 2020 | 121 | 2178 | 92 (25-333) | 0 (0-174) | 0 (0-174) |  |
| Hemoam, Manaus | February 2020 | 92 | 1656 | 0 (0-227) | 0 (0-227) | 0 (0-227) |  |
| Hemoam, Manaus | March 2020 | 92 | 1656 | 0 (0-227) | 0 (0-227) | 0 (0-227) |  |
| Hemoam, Manaus | November 2020 | 109 | 1962 | 51 (9-285) | 0 (0-192) | 0 (0-192) |  |
| Hemoam, Manaus | December 2020 | 92 | 1656 | 0 (0-227) | 0 (0-227) | 0 (0-227) |  |
| Hemoam, Manaus | January 2021 | 115 | 2070 | 146 (50-426) | 0 (0-182) | 48 (9-271) |  |

Supplementary Table 2. Incidence of infection in blood donors and reported case rates by month

| **Hemocenter** | **Month** | **DENV** | | **CHIKV** | | **ZIKV** | |
| --- | --- | --- | --- | --- | --- | --- | --- |
|  |  | **Incidence (95% CI)**  **infections/100PM** | **Case rate**  **/100,000** | **Incidence (95% CI)**  **infections/100PM** | **Case rate**  **/100,000** | **Incidence (95% CI)**  **infections/100PM** | **Case rate**  **/100,000** |
| Fundação Pro-Sangue, São Paulo | November 2019 | 0.19 (0.01-0.92) | 1.18 | 0 (0-0.75) | 0.28 | 0 (0-0.66) | 0.19 |
| Fundação Pro-Sangue, São Paulo | December 2019 | 0 (0-0.46) | 1.22 | 0 (0-0.70) | 0.25 | 0 (0-0.61) | 0.09 |
| Fundação Pro-Sangue, São Paulo | January 2020 | 0 (0-0.54) | 3.84 | 0 (0-0.80) | 0.58 | 0 (0-0.72) | 0.24 |
| Fundação Pro-Sangue, São Paulo | February 2020 | 0.20 (0.02-1.01) | 5.21 | 0 (0-0.79) | 0.6 | 0 (0-0.68) | 0.32 |
| Fundação Pro-Sangue, São Paulo | March 2020 | 0 (0-0.45) | 4.46 | 0 (0-0.67) | 0.46 | 0 (0-0.60) | 0.22 |
| Fundação Pro-Sangue, São Paulo | November 2020 | 0 (0-0.55) | 0.27 | 0 (0-0.80) | 0.13 | 0 (0-0.69) | 0.06 |
| Fundação Pro-Sangue, São Paulo | December 2020 | 0 (0-0.43) | 0.68 | 0 (0-0.63) | 0.29 | 0 (0-0.55) | 0.07 |
| Fundação Pro-Sangue, São Paulo | January 2021 | 0 (0-0.53) | 0.98 | 0 (0-0.79) | 0.09 | 0 (0-0.68) | 0.03 |
| Fundação Pro-Sangue, São Paulo | February 2021 | 0 (0-0.55) | 2.99 | 0 (0-0.80) | 0.21 | 0 (0-0.70) | 0.07 |
| Fundação Pro-Sangue, São Paulo | March 2021 | 0 (0-0.44) | 9.04 | 0 (0-0.64) | 0.58 | 0 (0-0.58) | 0.1 |
| Hemoribeirão, Ribeirão Preto | November 2019 | 0 (0-0.72) | 24.68 | 0 (0-1.06) | 0.32 | 0 (0-0.90) | 0.14 |
| Hemoribeirão, Ribeirão Preto | December 2019 | 0.54 (0.05-1.92) | 45.37 | 0 (0-1.06) | 0.37 | 0 (0-0.90) | 0.56 |
| Hemoribeirão, Ribeirão Preto | January 2020 | 0.32 (0.03-1.14) | 327.98 | 0 (0-0.64) | 1.24 | 0 (0-0.53) | 1.52 |
| Hemoribeirão, Ribeirão Preto | February 2020 | 0 (0-0.57) | 629.65 | 0 (0-0.80) | 1.15 | 0 (0-0.73) | 2.48 |
| Hemoribeirão, Ribeirão Preto | March 2020 | 0.99 (0.22-2.61) | 484.06 | 0 (0-0.65) | 1.33 | 0 (0-0.55) | 1.2 |
| Hemoribeirão, Ribeirão Preto | November 2020 | 0 (0-0.53) | 7.13 | 0 (0-0.80) | 0.6 | 0 (0-0.69) | 0.23 |
| Hemoribeirão, Ribeirão Preto | December 2020 | 0.16 (0.01-0.79) | 27.55 | 0 (0-0.63) | 0.69 | 0 (0-0.53) | 0.37 |
| Hemoribeirão, Ribeirão Preto | January 2021 | 0.20 (0.02-1.01) | 18.15 | 0 (0-0.79) | 0.27 | 0 (0-0.70) | 0.18 |
| Hemoribeirão, Ribeirão Preto | February 2021 | 0.41 (0.04-1.45) | 46.74 | 0 (0-0.79) | 0.32 | 0 (0-0.69) | 0 |
| Hemoribeirão, Ribeirão Preto | March 2021 | 0.82 (0.14-2.22) | 76.70 | 0 (0-0.64) | 0.64 | 0 (0-0.55) | 0.14 |
| **Hemocenter** | **Month** | **DENV** | | **CHIKV** | | **ZIKV** | |
|  |  | **Incidence (95% CI)**  **infections/100PM** | **Case rate**  **/100,000** | **Incidence (95% CI)**  **infections/100PM** | **Case rate**  **/100,000** | **Incidence (95% CI)**  **infections/100PM** | **Case rate**  **/100,000** |
| Hemominas, Belo Horizonte | November 2019 | 0 (0-0.51) | 4.38 | 0 (0-0.73) | 0.48 | 0 (0-0.66) | 0.32 |
| Hemominas, Belo Horizonte | December 2019 | 0 (0-0.57) | 5.41 | 0 (0-0.84) | 0.44 | 0 (0-0.76) | 0.04 |
| Hemominas, Belo Horizonte | January 2020 | 0 (0-0.47) | 18.56 | 0 (0-0.67) | 0.87 | 0 (0-0.59) | 0.59 |
| Hemominas, Belo Horizonte | February 2020 | 0 (0-0.58) | 45.57 | 0 (0-0.89) | 0.83 | 0 (0-0.76) | 0.52 |
| Hemominas, Belo Horizonte | March 2020 | 0 (0-0.52) | 70.47 | 0 (0-0.77) | 1.27 | 0 (0-0.68) | 0.36 |
| Hemominas, Belo Horizonte | November 2020 | 0 (0-0.54) | 6.42 | 0 (0-0.82) | 0.32 | 0 (0-0.71) | 0.08 |
| Hemominas, Belo Horizonte | December 2020 | 0 (0-0.43) | 6.74 | 0 (0-0.65) | 0.79 | 0 (0-0.56) | 0.04 |
| Hemominas, Belo Horizonte | January 2021 | 0 (0-0.55) | 7.15 | 0 (0-0.82) | 0.63 | 0 (0-0.71) | 0.2 |
| Hemominas, Belo Horizonte | February 2021 | 0 (0-0.58) | 7.71 | 0 (0-0.85) | 0.79 | 0 (0-0.72) | 0.12 |
| Hemominas, Belo Horizonte | March 2021 | 0 (0-0.45) | 10.63 | 0 (0-0.67) | 0.63 | 0 (0-0.56) | 0 |
| Hemorio, Rio de Janeiro | November 2019 | 0 (0-0.56) | 2.92 | 0 (0-0.82) | 4.09 | 0 (0-0.71) | 0.27 |
| Hemorio, Rio de Janeiro | December 2019 | 0 (0-0.60) | 4.32 | 0 (0-0.87) | 3.2 | 0 (0-0.75) | 0.21 |
| Hemorio, Rio de Janeiro | January 2020 | 0 (0-0.55) | 5.07 | 0 (0-0.81) | 5.34 | 0 (0-0.73) | 0.31 |
| Hemorio, Rio de Janeiro | February 2020 | 0 (0-0.51) | 3.13 | 0 (0-0.80) | 2.93 | 0 (0-0.68) | 0.27 |
| Hemorio, Rio de Janeiro | March 2020 | 0 (0-0.46) | 2.65 | 0 (0-0.70) | 2.49 | 0 (0-0.59) | 0.12 |
| Hemorio, Rio de Janeiro | November 2020 | 0 (0-0.53) | 0.95 | 0 (0-0.80) | 0.79 | 0 (0-0.68) | 0.04 |
| Hemorio, Rio de Janeiro | December 2020 | 0 (0-0.43) | 1.33 | 0 (0-0.64) | 1.53 | 0 (0-0.56) | 0.15 |
| Hemorio, Rio de Janeiro | January 2021 | 0 (0-0.55) | 0.65 | 0 (0-0.79) | 0.19 | 0 (0-0.68) | 0.06 |
| Hemorio, Rio de Janeiro | February 2021 | 0 (0-0.52) | 0.90 | 0 (0-0.80) | 0.3 | 0 (0-0.69) | 0 |
| Hemorio, Rio de Janeiro | March 2021 | 0 (0-0.54) | 1.58 | 0 (0-0.80) | 0.52 | 0 (0-0.69) | 0.06 |
| **Hemocenter** | **Month** | **DENV** | | **CHIKV** | | **ZIKV** | |
|  |  | **Incidence (95% CI)**  **infections/100PM** | **Case rate**  **/100,000** | **Incidence (95% CI)**  **infections/100PM** | **Case rate**  **/100,000** | **Incidence (95% CI)**  **infections/100PM** | **Case rate**  **/100,000** |
| Hemope, Recife | November 2019 | 0 (0-0.53) | 24.00 | 0 (0-0.78) | 7.66 | 0 (0-0.69) | 0.49 |
| Hemope, Recife | December 2019 | 0.22 (0.02-1.11) | 12.09 | 0 (0-0.87) | 4.62 | 0 (0-0.73) | 0.67 |
| Hemope, Recife | January 2020 | 0 (0-0.47) | 11.67 | 0 (0-0.67) | 6.05 | 0 (0-0.61) | 1.03 |
| Hemope, Recife | February 2020 | 0 (0-0.57) | 8.77 | 0 (0-0.85) | 3.69 | 0 (0-0.73) | 0.85 |
| Hemope, Recife | March 2020 | 0 (0-0.56) | 8.47 | 0 (0-0.82) | 2.12 | 0 (0-0.70) | 0.36 |
| Hemope, Recife | November 2020 | 0 (0-0.54) | 9.68 | 0 (0-0.79) | 6.77 | 0 (0-0.68) | 0.36 |
| Hemope, Recife | December 2020 | 0 (0-0.46) | 8.29 | 0 (0-0.68) | 4.66 | 0 (0-0.56) | 0.18 |
| Hemope, Recife | January 2021 | 0 (0-0.53) | 7.04 | 0.36 (0.03-1.40) | 8.13 | 0 (0-0.69) | 0.42 |
| Hemope, Recife | February 2021 | 0 (0-0.54) | 11.68 | 0.36 (0.03-1.42) | 9.33 | 0 (0-0.71) | 0.24 |
| Hemope, Recife | March 2021 | 0.53 (0.06-1.58) | 17.04 | 0 (0-0.69) | 21.37 | 0 (0-0.58) | 1.57 |
| Hemoam, Manaus | November 2019 | 0 (0-0.60) | 20.04 | 0 (0-0.90) | 0.48 | 0 (0-0.81) | 0.41 |
| Hemoam, Manaus | December 2019 | 0.20 (0.02-1) | 13.34 | 0 (0-0.78) | 0.37 | 0 (0-0.70) | 0.89 |
| Hemoam, Manaus | January 2020 | 0.31 (0.03-1.11) | 11.44 | 0 (0-0.61) | 0.11 | 0 (0-0.53) | 0.48 |
| Hemoam, Manaus | February 2020 | 0 (0-0.54) | 3.87 | 0 (0-0.79) | 0.26 | 0 (0-0.71) | 0.45 |
| Hemoam, Manaus | March 2020 | 0 (0-0.55) | 7.60 | 0 (0-0.80) | 0.52 | 0 (0-0.70) | 0.45 |
| Hemoam, Manaus | November 2020 | 0.17 (0.01-0.86) | 11.02 | 0 (0-0.68) | 0.41 | 0 (0-0.57) | 0.28 |
| Hemoam, Manaus | December 2020 | 0 (0-0.54) | 22.66 | 0 (0-0.78) | 0.17 | 0 (0-0.71) | 0.45 |
| Hemoam, Manaus | January 2021 | 0.49 (0.05-1.52) | 33.79 | 0 (0-0.63) | 0.79 | 0.15 (0.01-1.02) | 0.52 |

Supplementary Figure 1. Prevalence of chikungunya virus RNAemic donations, incidence of infections in blood donors, and reported case rates at six Brazilian blood centers

Supplementary Figure 2. Prevalence of Zika virus RNAemic donations, incidence of infections in blood donors, and reported case rates at six Brazilian blood centers


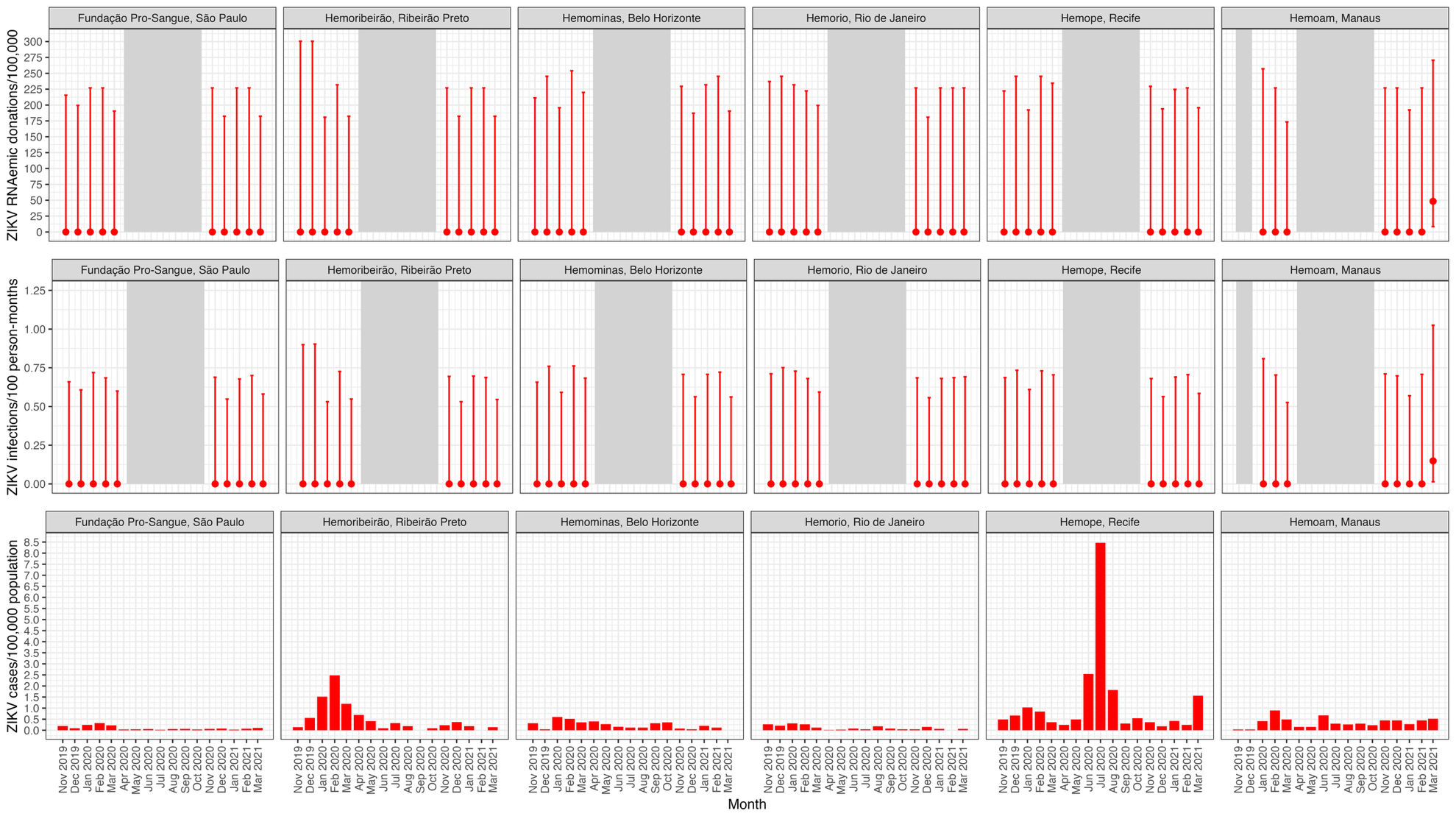

Supplement: Supplementary file 1 — Table S1. Minipool samples and donations tested, and prevalence of RNAemic donations by month. Table S2. Incidence of infection in blood donors and reported case rates by month. Figure S1. Prevalence of chikungunya virus RNAemic donations, incidence of infections in blood donors and reported case rates at six Brazilian blood centres. Figure S2. Prevalence of Zika virus RNAemic donations, incidence of infections in blood donors and reported case rates at six Brazilian blood centres. [file VOX-121-196-s001.docx]
